# Evaluation of the bond strength, surface roughness and wettability between 3D-printed denture base resin to denture lining material to different surface treatments

**DOI:** 10.4317/jced.62350

**Published:** 2025-03-01

**Authors:** Ana Larisse Carneiro Pereira, Bárbara Beatriz Teixeira Lima Cardoso de Souza, Larissa Mendonça de Miranda, Míria Rafaelli Souza Curinga, Lucas Cavalcante de Sousa, Edson Érick Fernandes de Queiroz, Valentim A. R. Barão, Adriana da Fonte Porto Carreiro

**Affiliations:** 1 PhD Candidate, Department of Dentistry, Federal University of Rio Grande do Norte (UFRN), Natal, Rio Grande do Norte, Brazil; 2 Research, Department of Dentistry, Federal University of Rio Grande do Norte (UFRN), Natal, Rio Grande do Norte, Brazil; 3 MSc Candidate, Department of Dentistry, Federal University of Rio Grande do Norte (UFRN), Natal, Rio Grande do Norte, Brazil; 4 DDS, Department of Dentistry, Federal University of Rio Grande do Norte (UFRN), Natal, Rio Grande do Norte, Brazil; 5 Associate Professor, Department of Prosthodontics and Periodontology, Piracicaba Dental School, Universidade Estadual de Campinas (UNICAMP), Piracicaba, São Paulo, Brazil; 6 Full Professor, Department of Dentistry, Federal University of Rio Grande do Norte (UFRN), Natal, Rio Grande do Norte, Brazil

## Abstract

**Background:**

The low bond strength between 3D-printed denture base resin and denture lining material is due to the difference in chemical structure, which justifies the use of methods to increase such bond. This study aims to examine the influence of different surface treatments on the bond strength, surface roughness, and wettability between 3D-printed denture resin and denture lining material.

**Material and Methods:**

Bar-shaped (75×10×10 mm) and square-shaped (10×10×3 mm) specimens were manufactured using heat-polymerized (HT) (VIPICRIL Plus) and 3D-printed (3D) (Prizma 3D Bio Denture) denture base resin. The bar specimens were sectioned, removing 3 mm from the center to facilitate the insertion of the denture lining material (Ufi Gel SC). Specimens were subjected to three surface treatments (n=15): no treatment (CT), immersion in monomer for 180 seconds (M), and airborne-particle abrasion oxide with 50 µm aluminum oxide (AP). The tensile bond strength was measured at a rate of 5mm/min before and after subjecting the specimens to thermocycling (10 000 cycles). The square-shaped specimens were used to assess average surface roughness (Ra) and wettability (°). Data analysis was performed using a 3-way ANOVA with Tukey’s post-test (*P*<.05).

**Results:**

The treatment of the 3D-printed denture base resin (1.200±0.486) with AP made the bond strength to the denture lining material similar to HT denture base resin (1.314±0.249), without the negative impact of aging. In contrast, M treatment increased the bond strength of both resins to the denture lining material (HT: 2.076±0.463; 3D: 1.534±0.484). Treatment with M provided a lower contact angle for the 3D and HT denture base resin, while the HT denture base resin presented a greater surface roughness for M and AP, compared to 3D.

**Conclusions:**

The 3D-printed denture base resin should undergo immersion in monomer treatment to enhance its bond strength with the denture lining material.

** Key words:**Denture lining material, Complete denture, Manufactured material, Denture base resin, Bond strength.

## Introduction

The use of denture lining material is a standard clinical practice that yields successful outcomes (1). Gradual alterations in oral tissues and ongoing resorption of the alveolar ridge necessitate relining of removable partial and complete dentures to enhance their fit and adaptation to the supporting tissues (1). This problem can be solved by the use of denture lining material, where the contact surface of the denture base resin with the tissues is covered with the denture lining material, promoting adaptation of the denture base to the remodeled tissues (2,3). This procedure reestablishes fit and retention of the denture and thus restores function, impacting the OHQoL (Oral Health-related Quality of Life) (4,5).

An effective bond between the denture lining material and the denture base resin is essential for optimal functionality (2). This bond is influenced by several factors, including the chemical composition and thickness of both the denture base resin and denture lining materials, the properties of the adhesive used, tear resistance, thermal stresses (6-10) and the polymers present in the denture base resin (11-14). A weak bond can harbor bacteria from promoting staining and delamination of the covering material, in addition to influencing the mechanical resistance of the denture lining base (13,15-17).

With the advent of CAD-CAM technology (computer-aided design and computer-aided manufacturing) and new materials, dentures can be fabricated using additive means (3D-printed denture resin) and subtractive (blocks and milling machines) (18). The materials used by subtractive manufacturing have a similar composition to materials produced from polymethylmethacrylate (PMMA). However, previous studies have shown that pre-polymerized PMMA blocks for milling dentures result in highly condensed and low porosity materials with chemical and mechanical properties superior to conventionally processed PMMA (18-21). When it comes to resins for lining CAD-CAM (3D printed) denture bases, there is inconsistency in terms of the bond strength between the two materials, justified by the difference in chemical composition of impression resins, when compared to milled and conventional materials, and denture lining material (3,22).

Previous literature shows that lining CAD-CAM (3D-printed) denture bases resin obtained lower bond strength values compared to resins for conventional and CAD-CAM (milled) denture bases resin (3,22). Other studies (13,23) have observed the effect of consistency (soft or rigid) and composition (resin or silicone) of denture lining material on bond strength to denture base resin. From this, they identified that silicone-based denture lining material produced greater bond strength for conventional and CAD-CAM (milled and 3D-printed) denture base resin (13). CAD-CAM lining resins (3D-printed) with rigid material demonstrated twice the bond strength compared to conventional and CAD-CAM (milled), while the soft material was not influenced by the type of denture base resin (23). In view of this, a single study proposed alternative surface treatments on denture (3D-printed), using tokuyama Rebase II (Tokuyama Dental Corp., Tokyo, Japan) normal adhesive, sandblasting, sandblasting and adhesive, sandblasting and silane, and Rocatec system (24). The authors showed that excellent adhesive strength can be obtained when the Rocatec system is applied to 3D-printed dentures base resin.

Therefore, a low evidence of new alternative surface treatments to increase the longevity of the union of these 3D-printed denture and denture lining material, justify carrying out this study. In this sense, the objective was to investigate the effect of different surface treatments on the bond strength, surface roughness and wettability between 3D-printed denture base resin and denture lining material.

## Material and Methods

**Figure 1:**
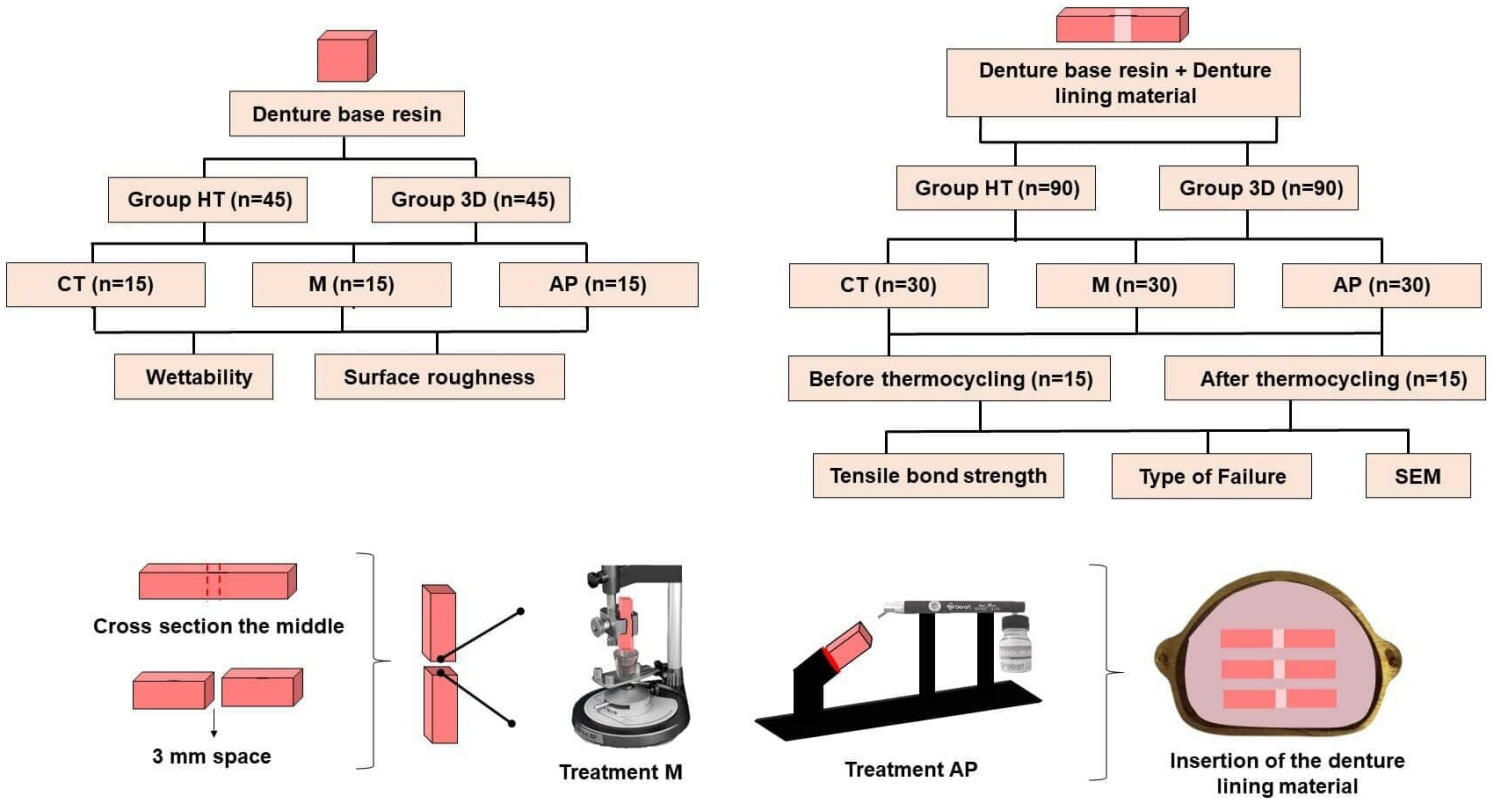
Study flowchart. HT: heat-polymerized denture base resin, 3D: 3D-printed denture base resin, CT: no surface treatment on denture base resin, M: immersion in monomer (180 seconds), AP: airborne-particle abrasion with aluminum oxide (50 µm), SEM: Scanning Electron Microscopy.

The experimental design is illustrated in Figure 1 and the materials used in the present study are detailed in  Table 1. One hundred and eighty specimens were manufactured following the ASTM International standards (D4762) to Flatwise Tensile Strength (D7291/D7291M) (25) with 75×10×10 mm (13) Bars were manufactured from two resins for denture bases: heat-polymerized denture base (VIPICRIL Plus; VIPI) (HP) and 3D-printed denture base (PRIZMA 3D Bio Denture/Makertech) (3D).

The bar manufactured from 3D-printed denture base resin were drawn in the 3D modeling program (Tinkercad/Autodesk) and saved in an 3D manufacturing formats (STL - Standart Tessellation Language). Before printing the bars, the calibrator indicated for each material was 3D-printed (Anycubic Photon Mono SE/Talmax) and measured with a digital calliper (MTX-316119/MTX) to ensure printer calibration. The STL file corresponding to the bar was sent to a 3D-printed denture base resin (angulation: vertical; bottom layers: 8; normal exposure time: 6.5 sec; layer thickness: 0.050 mm; bottom exposure time: 80.000) and then subjected to the post -processing process (washing in isopropyl alcohol for 5 minutes and then cured into an ultraviolet light chamber for 20 minutes).

**Figure 2:**
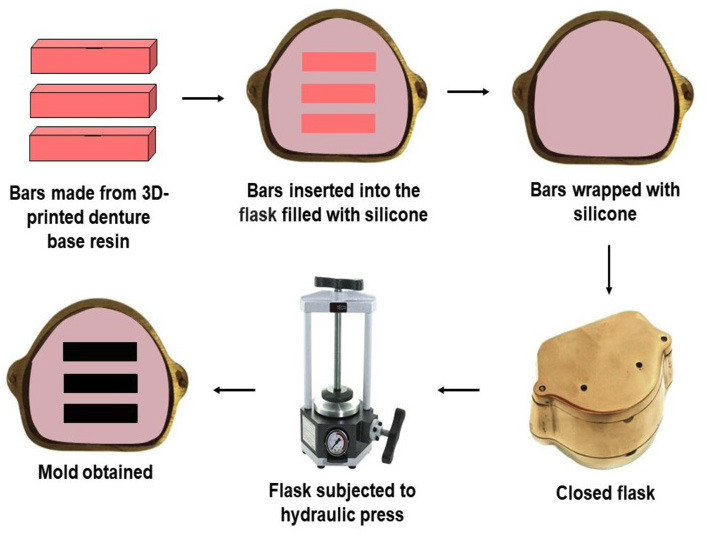
Sequence for making molds for inserting heat-polymerized denture base resin.

A flask furnace (Mac Dental) was filled with condensation silicone material (Zetalabor/Zhermack) and the 3D-printed denture base resin bars were inserted to create a negative impression with the initial dimensions of the bar (Fig. 2). This mold was used to manufacture bars from heat-polymerized polymethylmethacrylate denture base resin using the following protocol (22). The polymer and monomer ratio of the denture base resin followed the manufacturer’s instructions, using 14 g of powder for 6.5 mL of liquid. The denture base resin was inserted into the slicone molds isolated with water-soluble alginate solution (Cel Lac/S.S. White). A polyethylene sheet was placed over the denture base resin, and an initial pressure of 850 kgf was applied for 5 minutes, and subsequently, a final pressure of 1,250 kgf was applied for 20 minutes. The classic denture base resin polymerization procedure was carried out in an automatic device (Thermotron/Thermotron Dental Products), set to a water cycle heated to 74°C for 9 hours (26).

After bars manufacturing, they were sectionized with a water-cooled diamond blade (South Bay Technology) in a cutting machine (Isomet 1000/Buehler) in the middle and 3 mm of the denture lining material was removed from the center of the bars, leaving them with dimension 72×10×10 mm (22). With the bars sectioned, they were cleaned with gauze soaked in 90% alcohol and then divided into three groups (n=15) based on the type of surface treatment: Control (CT): without any treatment on the denture base resin; Monomer immersion (M): immersion in heat-polymerizable monomer (Vipi Cril Plus) for 180 seconds (the parts of the bar were separately fixed to a clamp attached to the movable vertical rod of the parallelometer [Bioart], allowing standardization of bar immersion in a container containing monomer [2 mm above the cutting limit] positioned on the eyeliner platform) (27), and airborne-particle abrasion oxide (AP) (50 µm aluminium oxide particles for 20 seconds, 2.5 bar, 90º inclination and 10 mm distance with a suspended particle abrasion instrument [Microjato Plus/Bioart]) (Fig. 1) (28).

After surface treatment, the bars were positioned in the existing molds in the flask, and the denture lining material was dispensed into the gap between the two parts of the bar (22), following the denture lining material manufacture insctructions: application of the adhesive (30 seconds), followed by the insertion of the denture lining material, and removal of excess with dental instruments.

Prior to all tests, the specimens were stored in distilled water at 37°C for 48 hours. Half of the specimens from each group were subjected to thermocycling (OMC 300 TSX/Odeme Dental Research), which consisted of a set of water baths of 5±1 ºC and 55±1 °C, dwell time of 30 seconds in each bath and 2 seconds out of the water between baths for 10 000 cycles (3).

**Figure 3:**
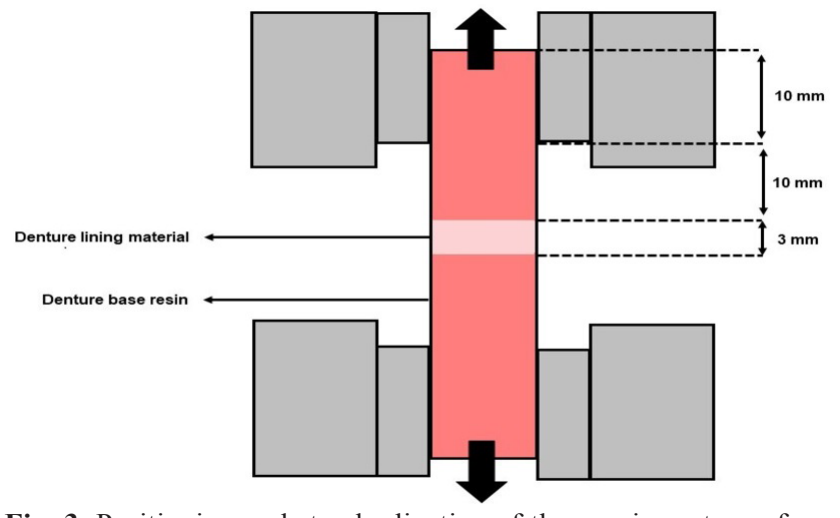
Positioning and standardization of the specimen to perform the tensile bond strength test.

The tensile bond strength between the denture base resin and denture lining material (before and after thermal cycling) was performed on a universal testing machine (Instron Model 4400 Universal Testing System/Instron Corp) at a crosshead speed of 5 mm/min (Fig. 3). Maximum tensile stress values before failure were recorded in Newtons (N). The load applied until fracture was obtained in N and converted to MPa using the following formula: σ (MPa) = L/A, where L is the load (N) for fracture between the denture base resin and denture lining material, and A is the interfacial area (mm2) (22).

The type of failure was classified as an adhesive (type 1), cohesive (type 2), or mixed mode (type 3), having to present at least 10% of the surface of the corresponding fracture pattern to be categorized (23), using a light microscope (EMF-1/Meiji Techno). An adhesive failure refers to a complete separation at the interface between the denture lining material and denture. A cohesive failure referred to a tear in the denture lining material, whereas a mixed failure had characteristics of both an adhesive and a cohesive failure (22). One sample per group was examined under the SEM (Scanning Electron Microscope) (JSM5600LV/JEOL Technics) to illustrate the failure mode of the optical microscope (13).

To understand the influence of surface treatment on the denture base resin, square-shaped (10×10×3 mm) specimens were manufactured for the heat-polymerizable and 3D-printed denture base resin. The manufacturing process followed the same steps previously mentioned for the bars.

The average surface roughness values Ra (µm) (n=15) of each sample were obtained using a precision roughness meter (Surtronic 25/Taylor Hobson), with a cut-off value of 0.80 mm. Three readings were taken on each sample, and the final value was obtained from an arithmetic mean between the three measurements, totaling 135 analyses for the 45 specimens (by denture resin) (29). The wettability test (n=15) was carried out according to the study of Sousa-Lima *et al*. (30), in which a drop (10 µL) of deionized water was dropped onto the surface of the samples from a distance of 20 mm with a graduated pipette (Peguepet CRAL/Cotia). After 90 seconds, a photograph was taken with a digital camera (Nikon DX AF-P NIKKOR/Nikon Corp) on a tripod 20 cm away. The right and left contact angles were measured using ImageJ software (ImageJ/U.S. National Institutes of Health), totaling 135 evaluations for all 45 specimens (by denture base resin).

The Kolmogorov-Smirnov test was used to test the normality of all data. To evaluate the effect of surface treatment (3 levels) on the tensile bond strength between the denture base (2 levels), surface treatments (3 levels), and time (2 levels [before and after thermocycling]), three-way ANOVA with Tukey’s post-test was performed to identify significant differences between the groups. In comparing the tensile bond strength between the surface treatments applied in the different experimental groups in relation to the control group, the Student’s t-test was used. A descriptive analysis was carried out for the type of failure using absolute and relative frequency (%). Two-way ANOVA was used to evaluate surface changes (wettability and surface roughness), with a post-test to test group by group. For all tests, *p*<0.05 was considered statistically significant.

## Results

Data relating to the comparison between surface treatments (CT [no surface treatment on denture base resin], M [immersion in monomer] and AP [airborne-particle abrasion with aluminium oxide 50 µm]) with the type of resin (HT [heat-polymerized denture base resin] and 3D [3D-printed denture base resin]) and time (before and after thermocycling) are shown in  Table 2 and  Table 3.

In the comparison between the types of resin within each surface treatment (*p*=0.013), when the HT resin surface received no treatment or was treated with the monomer, it obtained greater tensile bond strength to the denture lining material than the 3D-printed denture base resin. When the denture base resin (HT and 3D) was treated with AP, there was no significant difference between the HT and 3D denture base resin. When comparing the surface treatments to each other (*p*=0.000), the three differed significantly, with treatment M being the one that achieved the highest tensile bond strength with the denture lining material for both resins. The surface of the HT and 3D denture base resin, when not subjected to any surface treatment, suffered a negative influence in both thermocycling times (*p*<0.001), which did not happen for the M surface treatments (HT: *p*=0.439; 3D: *p*=0.158) and AP (HT: *p*=0.500; 3D: *p*=0.227).

**Figure 4:**
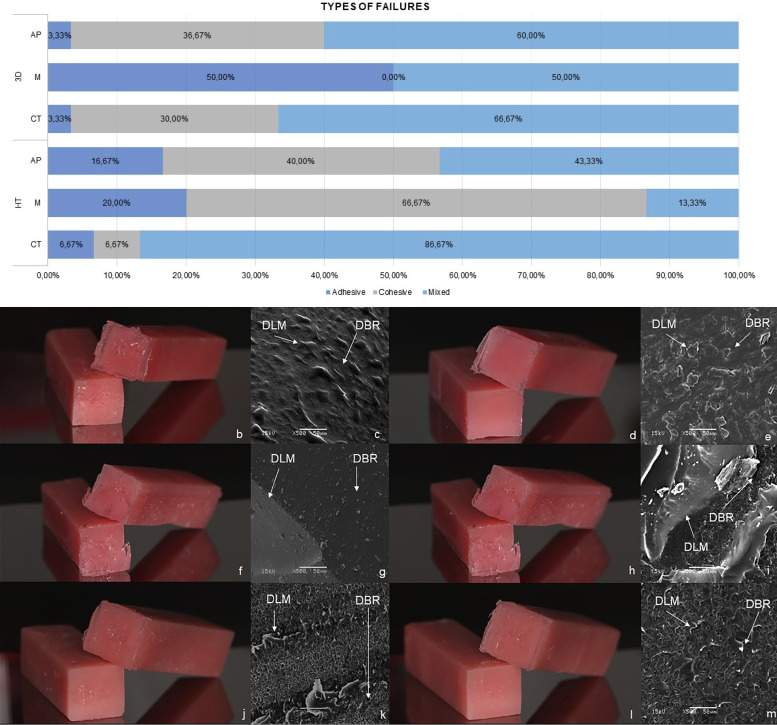
Types of failures at the interface between the denture base resin (DBR) and denture lining material (DLM). A, Failure type frequency. B, C No surface treatment of the heat-polymerized denture base resin (HT). D, E Surface treatment of the heat-polymerized denture base resin with immersion in monomer. F, G Surface treatment of the heat-polymerized denture base resin with airborne-particle abrasion with aluminum oxide (50 µm). H, I No surface treatment of the 3D-printed denture base resin (3D). J, K Surface treatment of 3D-printed denture base resin with monomer in immersion. L, M Surface treatment of the 3D-printed denture base resin with airborne-particle abrasion with aluminum oxide (50 µm).

The flaws that affected the interface between the resins for the denture and denture lining material they were influenced by surface treatment and thermocycling (Fig. 4). When the resins were treated with AP, mixed and cohesive type failures for HT and mixed for 3D were recorded. However, when the resins were treated with M, cohesive failures for HT and adhesive and mixed failures for 3D affected the interface with the denture lining material. When the resins did not receive any treatment, mixed-type failures were identified for HT and 3D denture base resin.

The impact of the type of surface treatment on the resins on wettability and surface roughness are shown in  Table 4 and  Table 5. Regarding wettability, when the resins were not subjected to any surface treatment, the 3D-printed denture base resin presented a higher angle of contact, in relation to HT resin. However, the surface treatment of resins with monomer obtained the lowest contact angle, differing significantly from the other surface treatments. The HT resin obtained higher surface roughness, compared to the 3D-printed denture base resin, for the three surface treatments.

## Discussion

This study reveals that the surface treatment of the resin for the heat-polymerized denture base resin with airborne-particle abrasion with aluminum oxide 50 µm and immersing the surface of 3D-printed denture base resin in monomer was able to increase the bond strength to the denture lining material.

The airborne-particle abrasion oxide removes impurities from the denture surface and improves mechanical bonding through increasing roughness and bonding area (31). It results in irregularities, valleys, depressions, many small pits, and scratches on the surface treated of the resin (32,33). Interestingly in this study, SEM investigation showed that sandblasted surfaces are rougher and debris-free and the surface roughness of the resin was increased for the heat-polymerized resin and 3D-printed denture. Therefore, blasting allowed the penetration of the denture lining material into the irregularities of the denture base resin, corroborating Park & Li (24).

Previous studies revealed that wetting the surfaces of the denture base resin was an effective approach to improving bonding with the denture lining material (27,28,34,35). The possible mechanism is that monomer was able to dissolve and smooth denture base materials, allowing the monomer to penetrate the surface, thus forming an interlocking network (34,35). A SEM analysis for the monomer experiment of the bonding surface of the heat-polymerized and 3D-printed denture with the denture veneering material showed that the monomer created a desmopolymerized surface that strengthened the bond between the two materials. This was also observed by Haghi *et al*. (27) and Kulkarni *et al*. (28) when stating that the surface treatment with immersion in monomer (180 seconds) of the denture resin increased the bond strength to the denture lining material.

Although the bond strength between the 3D-printed denture base resin and denture lining material increased when compared to other surface treatments, when immersed in monomer, it did not generate major morphological changes to the surface. This indicated that the 3D-printed denture base resin exhibited better chemical stability to the monomer than conventional denture base materials (36) This result can be attributed to the fact that the base material of the prosthesis for 3D printing is mainly composed of dimethacrylate monomers and oligomers (monomer-free composition) (37). To effectively etch the 3D-printed denture base resin, more aggressive organic solvents can be considered, such as acetone, chloroform, methylene chloride, and dichloromethane (38,39). Furthermore, 3D-printed denture base resin are fabricated using a layer-by-layer stacking method to prevent the formation of bubbles and voids on the surface. In contrast, bubbles and voids can form during the polymerization process of the denture base, leading to an increased surface roughness (40).

In the case of 3D-printed denture base resin, the layer-by-layer deposition process inherently results in a rougher surface, albeit one that can be minimized with the standardization of 3D printing parameters (41,42). Previous studies have demonstrated that adjustments to printing parameters, layer direction relative to the specimen surfaces, and printing orientations can mitigate surface roughness and even morphological changes in 3D-printed denture base resin (43).

Studies that highlight the relationship between resin viscosity and surface properties are still absent from the literature. However, it is already known that the viscosity of resin is also considered an important factor for successful production of DLPs (Digital Light Processing) (45,46). The viscosity of polymethyl methacrylate (PMMA) based resin for additive manufacturing increases with increasing PMMA content (45). Researchers revealed that the PMMA based resin with over 50wt% of its content was unsuitable for stereolithography due to its high viscosity (46). Furthermore, Lee *et al*. (44) showed that resin viscosity affects the influence of layer thickness and build angle on the hardness, flexural strength, and trueness of DLP-generated denture bases.

The contact angle can indirectly influence the bond strength. A larger contact angle indicates hydrophobic properties and consequently, lower wettability, while a smaller angle means hydrophilic properties and better wettability (47). Prosthetics often come into contact with saliva and water in clinical settings. Strong wettability implies water absorption, which can cause the prosthesis to swell and distort, leading to tensions at the interface between the covering material and the base of the prosthesis. This effect has an adverse impact on bond strength (48,6). This means that clinically sandblasting would have a positive effect for the bond between the printed denture base resin and denture lining material, since higher contact angles were measured.

Aging had a negative impact when the resin surface was not subjected to any treatment, which highlighted the importance of the surface treatments proposed by this study. The reduction in the bond strength is the result of swelling and stress build-up at the bond interface or of the changed viscoelastic properties of the resilient lining material, which renders the material stiffer and transmits the external loads to the bond site (44). Alternatively, it may be a result of the effects of fatigue and bond rupture from thermocycling-induced repetitive expansion and contraction of the polymer network (37).

According to Kawano *et al*. (6) denture lining material with a bond strength of 0.44 MPa is considered accepTable for clinical use. However, clinically one must consider the time of clinical use of the denture lining material, oral conditions, the presence of excessive forces on the base of the denture, and the maintenance of occlusal balance. All of these patient-centric factors can impact the bond strength between the denture and denture lining material, regardless of the type of resin or surface treatment.

This study is limited to being *in vitro* and having evaluated only one denture lining material and denture base resin. The results of this study indicated that the tested surface treatments can be applied to other denture lining material (based on silicone and acrylic resin, soft and hard) and other denture base resin (heat-polymerized, milled, and 3D-printed) to verify expanding or limiting applicability. Furthermore, clinical studies are needed to evaluate the effectiveness of the surface treatments tested by this study on denture base resin for subsequent lining.

## Conclusions

Based on the results found, it can be concluded that:

• The surface treatment of the heat-polymerized denture base resin with airborne-particle abrasion with aluminum oxide (50 µm) increased the bond strength to the denture lining material, without the negative effect of aging;

• Immersion in monomer (180 seconds) was able to increase the bond strength between 3D-printed denture base resin with the denture lining material, without the negative effect of aging;

• The absence of treatment on the surface of both resins promoted a greater reduction in bond strength with the denture lining material after aging, compared to the surface treatments tested.

## Figures and Tables

**Table 1:** Materials used in the study.

Acronyms	Commercial name	Composition	Manufacturer
HT	VIPICRIL Plus	Polymer (polymethyl methacrylate, polypropylene, pigments), monomer (methylmethacrylate, EDMA – crosslink, inhibitor)	VIPI
3D	PRIZMA 3D Bio Denture	Proprietary acrylate monomers (>10%), pigmentation and filler (≤10%), proprietary acrylate oligomers (<65%), diphenyl (2,4,6-trimethylbenzoyl)-phosphine oxide (<5%)	Makertech
-	Ufi Gel SC	Ufi Gel adesivo: Butanone; Ufi Gel SC: Polysiloxane	VOCO GmbH

HT: heat-polymerized denture base resin, 3D: 3D-printed denture base resin.

**Table 2:** Three-way ANOVA results for tensile bond strength.

	Sum of Squares	df	Mean Square	F-value	p-value
Surface treatments	9.266	2	4.633	26.439	0.000
Type of resin	6.354	1	6.354	36.261	0.000
Thermocycling	0.986	1	0.986	5.625	0.019
Surface treatments * Type of resin	1.575	2	0.788	4.494	0.013
Surface treatments * Thermocycling	2.140	2	1.070	6.106	0.003
Type of resin * Thermocycling	0.006	1	0.006	0.033	0.856
Surface treatments * Type of resin * Thermocycling	0.778	2	0.389	2.219	0.112
Error	29.440	168	0.175		
Overall	457.949	180			
Corrected Overall	50.545	179			
a. R Squared=.418 (Adjusted R Squared=.379)					

**Table 3:** Mean values and standard deviations of tensile bond strength between denture base resin and denture lining material.

Tensile bond strength (MPa)					
Surface treatments	Type of resin		Thermocycling		Overall
		N	Before	After	
CT	HT	15	1.884 ± 0.332	1.489 ± 0.332	1.686 ± 0.379 A
	3D	15	1.470 ± 0.427	.960 ± 0.463	1.215 ± 0.508 B
	Overall	30	1.677 ± 0.430	1.225 ± 0.475	1.451 ± 0.504 a
M	HT	15	2.135 ± 0.467	2.016 ± 0.467	2.076 ± 0.463 A
	3D	15	1.426 ± 0.559	1.642 ± 0.384	1.534 ± 0.484 B
	Overall	30	1.780 ± 0.621	1.829 ± 0.461	1.805 ± 0.543 b
AP	HT	15	1.262 ± 0.245	1.366 ± 0.251	1.314 ± 0.249 A
	3D	15	1.292 ± 0.304	1.107 ± 0.615	1.200 ± 0.486 A
	Overall	30	1.277 ± 0.272	1.236 ± 0.480	1.257 ± 0.387 c
Overall	HT	30	1.760 ± 0.511	1.624 ± 0.451	1.692 ± 0.484
	3D	30	1.396 ± 0.439	1.236 ± 0.568	1.316 ± 0.511
	Overall	60	1.578 ± 0.508	1.430 ± 0.546	1.504 ± 0.531

CT: no surface treatment on denture base resin, M: immersion in monomer (180 seconds), AP: airborne-particle abrasion with aluminium oxide (50 µm), HT: heat-polymerized denture base resin, 3D: 3D-printed denture base resin. Different letters indicate *p*<0.05. Capital letters: post sidak test comparing the type of resin (HT and 3D) within each surface treatments (CT, M and AP). Lowercase: Sidak post-test comparing surface treatments to each other.

**Table 4:** Two-way ANOVA results for wettability (º) and surface roughness (Ra).

	Wettability (º)^1^				
	Sum of Squares	df	Mean Square	F	p
Surface treatments	1848.844	2	924.422	5.026	0.009
Type of resin	883.913	1	883.913	4.806	0.031
Surface treatments * Type of resin	534.338	2	267.169	1.453	0.240
Error	15448.943	84	183.916		
Overall	1055158.943	90			
Corrected Overall	18716.038	89			
	Surface Roughness (Ra)^2^				
	Sum of Squares	df	Mean Square	F	p
Surface treatments	77.649	2	38.824	71.636	0.000
Type of resin	59.927	1	59.927	110.573	0.000
Surface treatments * Type of resin	39.708	2	19.854	36.633	0.000
Error	45.525	84	.542		
Overall	496.129	90			
Corrected Overall	222.809	89			

1. R Squared =0.175 (Adjusted R Squared = 0.125) 2. R Squared =0.796 (Adjusted R Squared = 0.784)

**Table 5:** Mean values ± standard deviations for wettability (º) and surface roughness (Ra) of denture base resin after being subjected to different surface treatments.

		Wettability (º)			Surface Roughness (Ra)		
Surface treatments	N	Type of resin		Overall	Type of resin		Overall
		HT	3D		HT	3D	
CT	15	104.44 ± 8.89 A	116.13 ± 7.18 B	110.28 ± 9.92 a	3.63 ± 1.20 A	0.12 ± 0.04 B	1.88 ± 1.97 a
M	15	100.97 ± 5.81 A	100.84 ± 9.55 A	100.90 ± 7.77 b	0.88 ± 0.50 A	0.20 ± 0.09 B	0.54 ± 0.49 b
AP	15	107.12 ± 28.17 A	110.44 ± 10.50 A	107.31 ± 14.50 a	3.15 ± 1.07 A	2.45 ± .63 B	2.80 ± 0.93 c
Overall	45	104.17 ± 17.17	110.44 ± 10.50	107.31 ± 14.50	2.55 ± 1.54	0.92 ± 1.14	1.74 ± 1.58

CT: no surface treatment on denture base resin, M: immersion in monomer (180 seconds), AP: airborne-particle abrasion with aluminum oxide (50 µm), HT: heat-polymerized denture base resin, 3D: 3D-printed denture base resin. Different letters indicate *p*<0.05. Capital letters: post sidak test comparing the type of resin (HT and 3D) within each surface treatments (CT, M and AP). Lowercase: Sidak post-test comparing surface treatments to each other.

## Data Availability

The datasets used and/or analyzed during the current study are available from the corresponding author.
